# Cancer Imaging Phenomics via CaPTk: Multi-Institutional Prediction of Progression-Free Survival and Pattern of Recurrence in Glioblastoma

**DOI:** 10.1200/CCI.19.00121

**Published:** 2020-03-19

**Authors:** Anahita Fathi Kazerooni, Hamed Akbari, Gaurav Shukla, Chaitra Badve, Jeffrey D. Rudie, Chiharu Sako, Saima Rathore, Spyridon Bakas, Sarthak Pati, Ashish Singh, Mark Bergman, Sung Min Ha, Despina Kontos, MacLean Nasrallah, Stephen J. Bagley, Robert A. Lustig, Donald M. O’Rourke, Andrew E. Sloan, Jill S. Barnholtz-Sloan, Suyash Mohan, Michel Bilello, Christos Davatzikos

**Affiliations:** ^1^ Center for Biomedical Image Computing and Analytics, Perelman School of Medicine, University of Pennsylvania, Philadelphia, PA; ^2^ Department of Radiology, Perelman School of Medicine, University of Pennsylvania, Philadelphia, PA; ^3^ Department of Radiation Oncology, Christiana Care Helen F. Graham Cancer Center and Research Institute, Newark, DE; ^4^ Department of Radiation Oncology, Perelman School of Medicine, University of Pennsylvania, Philadelphia, PA; ^5^ Department of Radiology, University Hospitals-Seidman Cancer Center, Cleveland, OH; ^6^ Case Western Reserve University School of Medicine, Cleveland, OH; ^7^ Case Comprehensive Cancer Center, Cleveland, OH; ^8^ Department of Radiology and Biomedical Imaging, University of California at San Francisco, San Francisco, CA; ^9^ Department of Pathology and Laboratory Medicine, Perelman School of Medicine, University of Pennsylvania, Philadelphia, PA; ^10^ Abramson Cancer Center, University of Pennsylvania, Philadelphia, PA; ^11^ Perelman School of Medicine, University of Pennsylvania, Philadelphia, PA; ^12^ Glioblastoma Translational Center of Excellence, Abramson Cancer Center, University of Pennsylvania, Philadelphia, PA; ^13^ Department of Neurologic Surgery, University Hospitals-Seidman Cancer Center, Cleveland, OH; ^14^ Department of Population and Quantitative Health Sciences, Case Western Reserve University School of Medicine, Cleveland, OH

## Abstract

**PURPOSE:**

To construct a multi-institutional radiomic model that supports upfront prediction of progression-free survival (PFS) and recurrence pattern (RP) in patients diagnosed with glioblastoma multiforme (GBM) at the time of initial diagnosis.

**PATIENTS AND METHODS:**

We retrospectively identified data for patients with newly diagnosed GBM from two institutions (institution 1, n = 65; institution 2, n = 15) who underwent gross total resection followed by standard adjuvant chemoradiation therapy, with pathologically confirmed recurrence, sufficient follow-up magnetic resonance imaging (MRI) scans to reliably determine PFS, and available presurgical multiparametric MRI (MP-MRI). The advanced software suite Cancer Imaging Phenomics Toolkit (CaPTk) was leveraged to analyze standard clinical brain MP-MRI scans. A rich set of imaging features was extracted from the MP-MRI scans acquired before the initial resection and was integrated into two distinct imaging signatures for predicting mean shorter or longer PFS and near or distant RP. The predictive signatures for PFS and RP were evaluated on the basis of different classification schemes: single-institutional analysis, multi-institutional analysis with random partitioning of the data into discovery and replication cohorts, and multi-institutional assessment with data from institution 1 as the discovery cohort and data from institution 2 as the replication cohort.

**RESULTS:**

These predictors achieved cross-validated classification performance (ie, area under the receiver operating characteristic curve) of 0.88 (single-institution analysis) and 0.82 to 0.83 (multi-institution analysis) for prediction of PFS and 0.88 (single-institution analysis) and 0.56 to 0.71 (multi-institution analysis) for prediction of RP.

**CONCLUSION:**

Imaging signatures of presurgical MP-MRI scans reveal relatively high predictability of time and location of GBM recurrence, subject to the patients receiving standard first-line chemoradiation therapy. Through its graphical user interface, CaPTk offers easy accessibility to advanced computational algorithms for deriving imaging signatures predictive of clinical outcome and could similarly be used for a variety of radiomic and radiogenomic analyses.

## INTRODUCTION

Cancers display hallmarks of spatial and temporal heterogeneity at various scales, contributing to unfavorable prognosis and treatment failure.^1^ Clinical imaging offers the possibility of elucidating multifaceted phenotypic aspects of cancer structure and physiology through acquisition of diverse modalities.^1-3^ Semantic features such as descriptors of size, morphology, and location that are commonly measured from radiologic images, are limited in revealing the underlying cancer heterogeneity.^4,5^ Cancer imaging phenomics (CIPh) is an emerging field for quantitative analysis of oncologic multiparametric imaging. Through mathematical measurements of the aforementioned features, commonly known as radiomic features, CIPh provides a broad spectrum of phenotypic imaging signatures, which potentially brings increased precision to diagnosis, prognosis, and prediction of response to therapy.^6,7^

CIPh signatures may play a key role in paving the path for precision medicine, as suggested by a growing body of studies over the past few years.^8-10^ But radiomic analysis of high-dimensional feature spaces, if they are not accompanied by high-throughput computational methods, could complicate the process of deriving conclusions for planning treatment in clinical settings.^11-15^ This limits the availability of CIPh signatures when designing clinical trials or applying them to patient-specific problems. In this context, the Cancer Imaging Phenomics Toolkit (CaPTk), an imaging analytics suite of open-source software utilities and algorithms, has been developed to enable the quick derivation of extensive sets of CIPh features for precision diagnosis and predictive modeling to support personalized cancer therapy.

**CONTEXT**

**Key Objective**
Describe how machine learning methods (radiomics) based on multiparametric magnetic resonance imaging (MRI) scans can aid in personalized prognosis and treatment planning in patients with glioblastoma.
**Knowledge Generated**
Using the Cancer Imaging Phenomics Toolkit (CaPTk) open-source software, we created quantitative signatures of progression-free survival and recurrence pattern in patients with glioblastoma. We showed the feasibility of a radiomics approach for the two aforementioned clinical applications in a multi-institutional analysis of 80 patients on the basis of preoperative MRI scans.
**Relevance**
Predictive radiomic models can be used to help in first-line decision making on a patient-specific basis.

CaPTk offers a systematic quantification platform for designing clinical research studies using radiomics and radiogenomics methods, from harmonized data preprocessing and extraction of rich sets of CIPh features that represent tumor characteristics to integration of features using appropriate machine learning (ML) methods for precision diagnosis and prediction.^7,11,14,16-18^ Herein we examine the application of CaPTk in glioblastoma multiforme (GBM), which is an aggressive and genetically diverse neoplastic malignancy with poor response to available therapies, rapid progression, inevitable recurrence, short median progression-free survival (PFS),^19^ limited treatment options, and a median overall survival (OS) of 15 months.^20^ The efficacy of novel treatment strategies is most objectively gauged by the improvement in patient’s OS or PFS^21^ (as a surrogate marker of OS in patients with GBM^22,23^). Although it is challenging to objectively define PFS, when it is used as an end point, it has the advantage of helping to complete trials faster and more rapidly determine which interventions may be helpful for patients.

Studies of PFS and OS have suggested the benefit of maximal surgical resection and adjuvant concurrent chemoradiation therapy to locally control recurrence in patients with GBM.^24^ Nonetheless, the majority of GBM tumors progress in proximity to the surgical resection cavity.^25^ This propensity to recurrence has been attributed to a GBM cancer subpopulation that is resistant to chemoradiation therapy, and recurrence disperses the tumor cells into the surrounding tissues or even to distant locations.^26^

Reliable upfront prediction of PFS and recurrence pattern (RP) may facilitate better personalization of treatment by, for example, stratifying patients into clinical trials for treatment intensification and/or supportive care and improving the efficiency of clinical trial design.^27^ In this article, we address the problem of constructing personalized prognostic signatures of GBM related to PFS and RP by leveraging CIPh signatures generated from the CaPTk platform.

## PATIENTS AND METHODS

### Study Design

**FIG 1.**
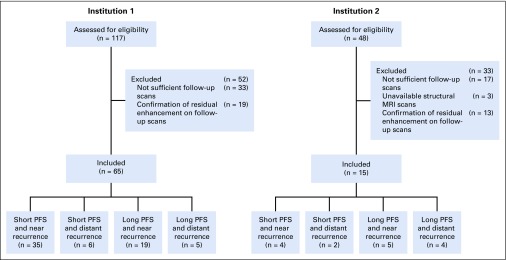
Representation of the data inclusion and exclusion process and analysis. MRI, magnetic resonance imaging; PFS, progression-free survival.

Data from patients with newly diagnosed GBM from two institutions (institution 1, Hospital of the University of Pennsylvania, n = 65; institution 2, Ohio Brain Tumor Study, n = 15^28,29^) were retrospectively collected after obtaining approval from the institutional review boards. Details about the inclusion and exclusion criteria and imaging parameters for both institutions are provided in the Data Supplement. Characteristics of the patients recruited in this study are provided in Table 1 and Figure 1.

**TABLE 1.**
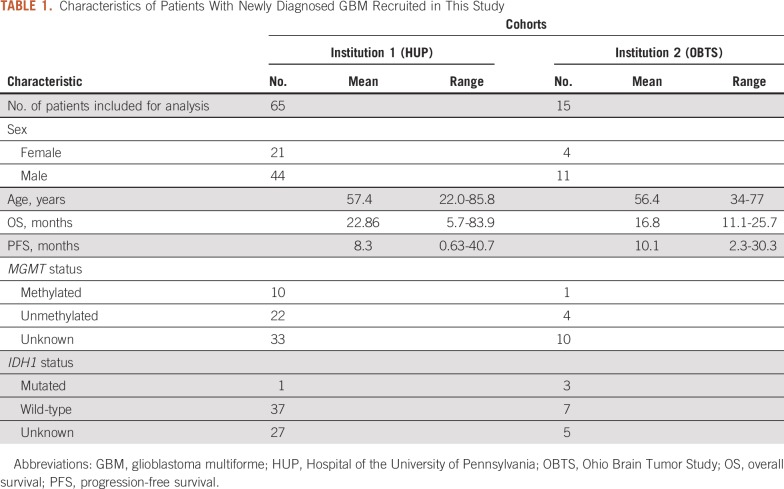
Characteristics of Patients With Newly Diagnosed GBM Recruited in This Study

PFS was measured from the date of initial diagnosis until tumor progression by neuroradiologists (J.D.R. and M.B. at institution 1 and C.B. at institution 2), in compliance with criteria for tumor progression provided by the Response Assessment in Neuro-Oncology Working Group.^30^ RP was determined by neuroradiologists (S.M. and M.B. at institution 1 and C.B. at institution 2) who were blinded to the patients’ clinical and genetic information. RP was defined on the basis of the distance between the surgical resection cavity and the new enhancing lesion on the T1-weighted contrast-enhanced (T1CE) scan at the recurrence time point: near recurrence was considered as a contiguous enhancement with the resection cavity, and distant recurrence was considered as an enhanced lesion that is noncontiguous with the cavity (with a nonenhancing region between the cavity and the new enhancement focus).

### Radiomic Analysis Using CaPTk

**FIG 2.**
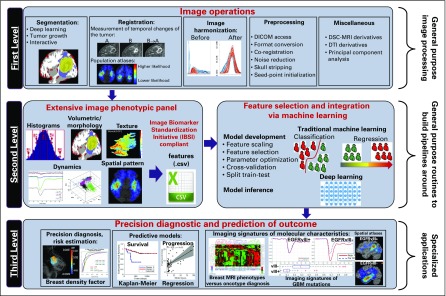
A schematic figure outlining the design and architecture of the Cancer Imaging Phenomics Toolkit (CaPTk). The software consists of three levels for radiomic analysis. DICOM, Digital Imaging and Communications in Medicine; DSC, dynamic susceptibility contrast-enhanced; DTI, diffusion tensor imaging; GBM, glioblastoma multiforme; MRI, magnetic resonance imaging.

The radiomic analysis for prediction of PFS and RP in this study was designed and carried out using CaPTk open-source software (https://www.med.upenn.edu/cbica/captk/). CaPTk was designed on the basis of a three-level functionality for radiomic analyses (Fig 2). The first level provides image preprocessing tasks, such as conversion of image formats, segmentation, registration, and smoothing. The second level comprises various general-purpose routines such as feature extraction, feature selection, and ML. These routines are used within CaPTk for specialized closed-form applications and are also available for customized analysis pipelines. In particular, this level targets extraction of various features that capture different aspects of local, regional, and global imaging patterns, which results in an extensive feature panel that is compliant with the guidelines provided by the Image Biomarker Standardization Initiative,^31^ thus ensuring reproducible and comparable radiomic features. It also synthesizes features to distinguish smaller more meaningful feature subsets from larger feature sets and uses ML to build predictive and diagnostic models. The third level of CaPTk focuses on integrating these features into specialized applications via ML algorithms to accomplish specific goals such as making precision diagnoses, assessing risk for developing cancer, and creating models that predict response and survival.

#### Image preprocessing.

Preprocessing of multiparametric magnetic resonance imaging (MP-MRI) data, including T1-weighted (T1w) contrast-enhanced (T1CE), T2-weighted (T2w), T2-fluid-attenuated inversion recovery (T2-FLAIR), diffusion tensor imaging (DTI), and dynamic susceptibility contrast-enhanced MRI (DSC-MRI) images, was performed with the CaPTk software (for details, see Figure 2 and the Data Supplement). The calculated perfusion derivatives for institution 1 comprised peak height (PH), percentage of signal recovery (PSR), and relative cerebral blood volume (rCBV). The diffusion derivatives included axial diffusivity (AX), trace (TR), radial diffusivity (RAD), and fractional anisotropy (FA).

Automated segmentation was performed with the DeepMedic^32^ module and was approved or revised by an experienced radiologist (S.M.) through CaPTk to identify tumoral subregions such as enhancing tumor (ET), nonenhancing portion of the tumor core (NC), and peritumoral edema (ED). Definitions can be found in Akbari et al^11^ and Bakas et al^33^. Two additional tumor subregions representing the tumor core (TC, defined as the union of ET and NC subregions) and whole tumor volume (WT, calculated as the union of all three tumor subregions [ie, ET, NC, and ED]) were also generated.

#### Radiomic feature extraction.

The preprocessed images were passed through the feature extraction panel of CaPTk. Relevant imaging features were computed for each patient from the five tumoral regions (ET, NC, ED, TC, and WT) and all modalities to capture phenotypic characteristics of short versus long PFS and near versus distant RP. The extracted features included volume, shape, and size; intensity; histogram; and gray-level co-occurrence matrix (GLCM) features. These features make it possible to quantitatively assess morphologic and spatial heterogeneity properties of the tumoral regions. A total of 1,980 features were extracted from data for institution 1, and 1,016 features were extracted for multi-institutional analysis.

#### Quantification schemes for predicting PFS and RP.

For predicting PFS, the patients were stratified into two classes: short PFS (≤ 8.3 months; institution 1, n = 41; institution 2, n = 6) and long PFS (> 8.3 months; institution 1, n = 24; institution 2, n = 9). The number of patients with near recurrence locations were n = 54 for institution 1 and n = 9 for institution 2; the number of patients with distant recurrence locations were n = 11 for institution 1 and n = 6 for institution 2.

The dimensionality of the feature space was reduced by using sequential forward feature selection and a support vector machine (SVM) model through 10-fold cross-validation to determine the feature combinations most predictive of PFS and RP status. Here, six experimental schemes were designed for assessing PFS (schemes 1 to 3) and RP (schemes 4 to 6) for single- and multi-institutional data.

##### Scheme 1 (PFS) or scheme 4 (RP) for institution 1 data.

The cohort of 65 patients from institution 1 was randomly partitioned into discovery (70% of the data) and replication (30% of the data) subsets over 50 iterations. The SVM model was trained using 10-fold cross-validation on the discovery subset and independently validated on the replication cohort.

##### Scheme 2 (PFS) or scheme 5 (RP) for institution 1 and institution 2 data: random discovery and replication cohorts.

This scheme was designed for an integrated cohort of 80 patients from both institutions. Selection of the discovery and replication cohorts was similar to that in scheme 1.

##### Scheme 3 (PFS) or scheme 6 (RP) for institution 1 (discovery cohort) and institution 2 (independent replication cohort).

Similar to previous schemes, the SVM model was trained using a 10-fold cross-validation on the discovery subset and tested on the replication data.

## RESULTS

### Classification Performance of the Predictive Models

**FIG 3.**
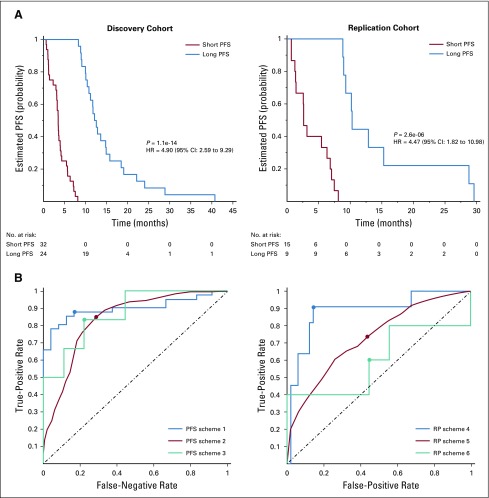
(A) Kaplan-Meier curves for predicting short and long progression-free survival (PFS) in the (left) discovery and (right) replication cohorts of multi-institutional data in scheme 2 (in one of the random partitioning iterations). (B) Analysis of classification performances using receiver operating characteristic curves for (left) PFS prediction based on schemes 1 to 3 and (right) recurrence pattern (RP) prediction on the basis of schemes 4 to 6.

Performance of our classification schemes in predicting short versus long PFS or near versus distant RP are presented in Table 2. Kaplan-Meier PFS curves from the discovery and replication sets stratified into short-PFS and long-PFS groups for one of the randomly partitioned iterations in scheme 2 are shown in Figure 3A. The receiver operating characteristic (ROC) curves for each of our predictive schemes for stratifying patients based on their PFS or RP are shown in Figure 3B. A list of the most frequently selected features is provided in the Data Supplement.

**TABLE 2.**
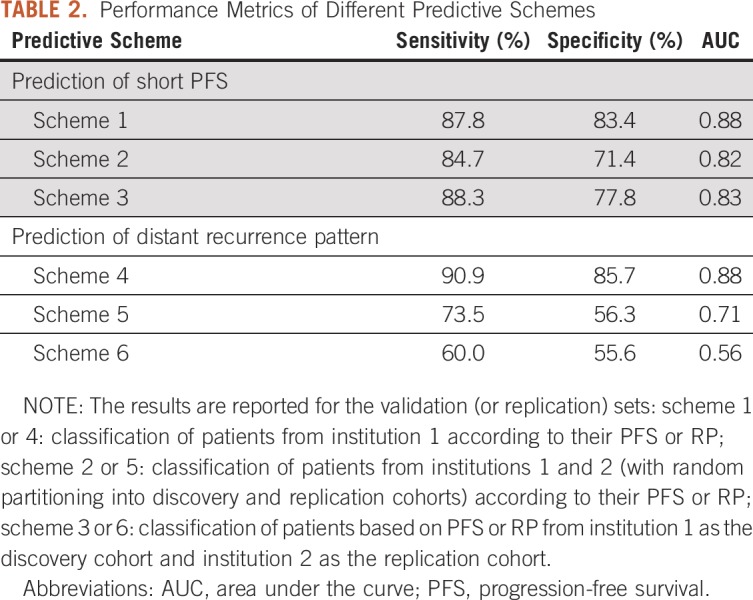
Performance Metrics of Different Predictive Schemes

#### Prediction of PFS.

**FIG 4.**
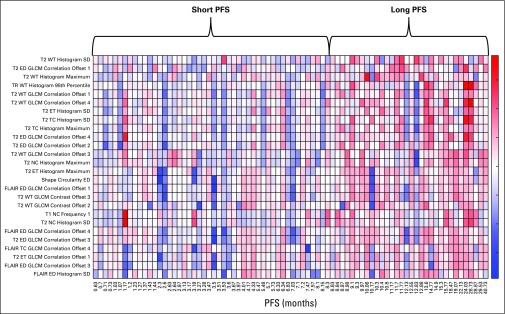
Heat map of the 26 top-ranked features most frequently selected for classification of multi-institutional data on the basis of progression-free survival (PFS) according to scheme 2. The *x*-axis represents the PFS values for each of the patients, and the *y*-axis shows the radiomic features. ED, edema; ET, enhancing tumor; FLAIR, fluid-attenuated inversion recovery; GLCM, gray-level co-occurrence matrix; NC, nonenhancing core; SD, standard deviation; T2, TC, tumor core; TR, trace; WT, whole tumor.

In scheme 1, the selected features mainly represent imaging markers of neo-angiogenesis yielded by increased mean PSR value within the ED region, mean rCBV value within ET, and cellular density indicated by lower values of AX or TR within the WT area for the short-PFS compared with the long-PFS group. In scheme 2, as the results in Table 2 suggest, the specificity of predicting PFS decreases when the features derived from perfusion or DTI imaging are unavailable. The top-ranked selected features were mainly among features indicating spatial heterogeneity of T2w images in ED or WT, shape measure of ED circularity, and TR values within the WT region, all of which were increased in the long-PFS category. Selected features for classification of patients in this scheme are presented in the heat map in Figure 4. In scheme 3, many of the selected features were similar to those in scheme 2.

#### Prediction of RP.

In scheme 4, by integrating features derived from advanced imaging (texture features from PH in NC region and PSR within ED, histogram and texture features from FA in ED or WT, texture features from FLAIR within NC, and shape measure of ED eccentricity), area under the curve (AUC) of 0.88 was achieved for discrimination of near RP from distant RP. Interestingly, in this scheme, FA within ED as an indirect measure of microstructural damage was critical in distinguishing the RP. In scheme 5, multivariable analysis of the RP based on multi-institutional data was suggestive of the importance of histogram and texture features computed from subtraction of T1w from T1CE (T1SUB) as a measure of permeability of tumor vasculature within ED and texture features within ED on T2w or T2-FLAIR images. This classification scheme resulted in AUC of 0.71. Because advanced imaging was unavailable, the sensitivity of this multivariable analysis was notably reduced. In scheme 6, the AUC for classification of near from distant recurrence in the independent data (institution 2) was 0.56. As with scheme 5, textural features within the ED region on T1SUB, T2w, and T2-FLAIR and shape measure of eccentricity within ED were among the selected features.

### Integrated Prediction of PFS and RP

**FIG 5.**
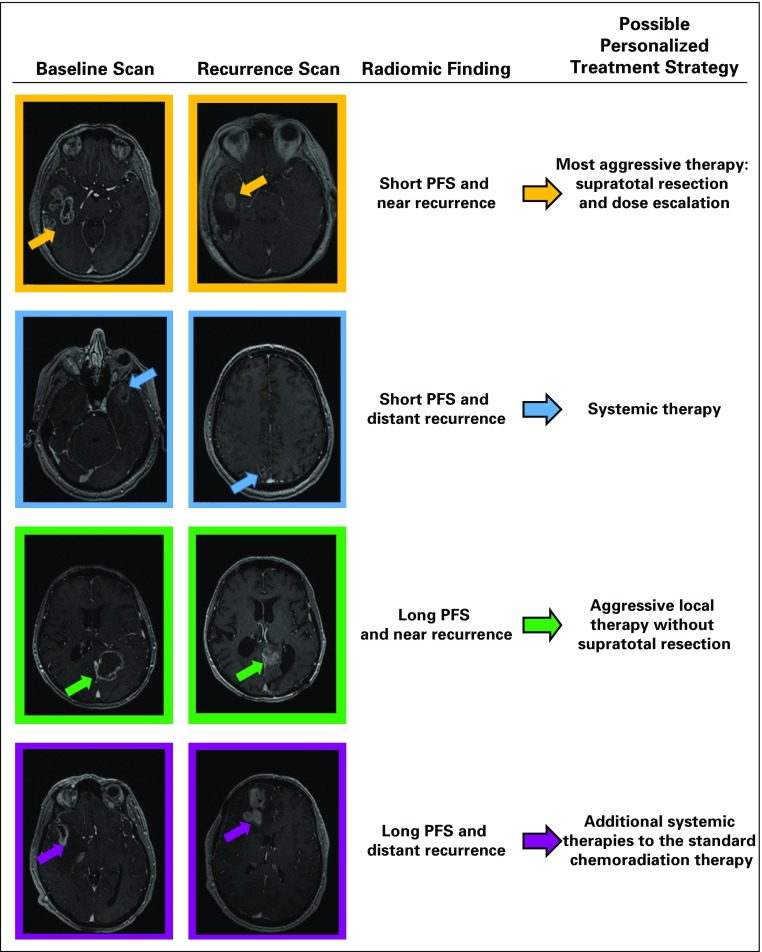
Examples of different schemes of progression-free survival (PFS) and recurrence pattern with possible therapy personalized treatment strategies: the first and second columns indicate the baseline and recurrence scans for each example, the radiomic finding for each example is displayed in the third column, and the fourth column shows the suggested personalized therapy plan for each example.

For upfront prediction of recurrence after primary resection for GBM tumors, the classifiers of schemes 1 and 4, 2 and 5, or 3 and 6 can be integrated. Figure 5 shows examples of patients with different combinations of time and location of recurrence (PFS and RP) and the possible treatment strategies that it may be helpful to consider for each patient.

## DISCUSSION

This study investigated the application of in vivo MP-MRI phenomic signatures leveraging ML and the CaPTk software suite for predicting PFS and RP in patients with GBM who received standard-of-care therapy and aiming to offer advanced imaging-based biomarkers for clinical decision making and personalized treatment planning.

We designed different multivariable predictive signatures based on MP-MRI for prediction of PFS or RP for single- or multi-institutional data. The selected radiomic features for predicting PFS in the patient cohort of institution 1 with available advanced MRI consisted mainly of intensity or histogram features calculated from PSR, AX, and TR maps. In the absence of advanced imaging, PFS was more reliably estimated by texture (GLCM) features computed from T2w images or TR maps within ED or WT regions. This finding suggests that more mathematically sophisticated features that better represent the spatial heterogeneity of the tumor from conventional MRI and diffusion weighted imaging may partly compensate for the lack of perfusion and other more advanced imaging measures. Nonetheless, TR in ED, a marker of diffusion restriction and infiltration of tumorous cells, played a critical role in stratification of patients on the basis of their PFS, with higher TR values in patients with longer PFS.

The importance of infiltrated and edematous peritumoral tissue in patients’ response to therapy has been shown in other studies.^11,34-36^ ED circularity, implying the roundness of the region, was among the high-ranked selected features, highlighting the importance of this feature for predicting PFS; patients with longer PFS show higher ED circularity. In previous studies, it has been reported that more circular peritumoral edema is associated with longer OS.^15,37^

When DTI features were included in the analysis, the most predictive features for near versus distant RP were chosen from FA maps within ED. FA may be considered an indirect marker of microstructural disruption of white matter as a consequence of tumor infiltration.^38^ Distinct spatial heterogeneity within peritumoral edema on FA maps between near versus distant RP might relate to infiltrated pathways that the tumor cells use for invading the distant regions. Near recurrence showed lower AX, RAD, and TR and higher FA values in the peritumoral edema region, implying higher diffusion restriction and microstructural damage in the near compared with the distant RP, as suggested before.^11,18^ It has been proposed that the tumor cell subpopulations with lower cell density that reside farther from the tumor core tend to progress into distant RP.^24^

In multi-institutional analysis of RP, texture features on T1SUB and T2w/T2-FLAIR were significantly discriminative, suggesting the importance of vascular permeability and tissue water and cell content. The shape measure of ED eccentricity was also among the top-ranked features; patients with distant recurrence had lower ED eccentricity.

Treatment strategies in the management of GBM are often a shared process between the health care providers and the patients, balancing the potential benefits and risks with the goals of the patient. The proposed CIPh predictive signatures can provide upfront information about the behavior of the disease after primary resection, which allows for a more personalized approach to treating each patient.

We propose that accurate prediction of PFS and RP may allow for more individualized treatment planning for patients with GBM, with one of the following possible options (it should be noted that these approaches need to be proven effective in well-designed clinical trials before they are routinely implemented in the clinic). First, for short PFS and near recurrence, aggressive local therapy or supratotal resection plus dose-escalated radiation therapy or other aggressive local therapies should be used for first-line treatment. Second, for short PFS and distant recurrence, patients should be recruited for trials that evaluate early use of aggressive systemic therapies, including immunotherapy or chemotherapy rather than aggressive local therapy. The standard of care for local therapy after surgery (radiation and temozolomide) may not provide much benefit for a patient with a high chance of rapid distant recurrence. The survival of such patients is generally less favorable, so physicians may be more inclined to pursue comfort or best supportive care rather than aggressive and expensive antineoplastic therapy that may cause toxicity without benefit. Third, for long PFS and near recurrence, patients should be recruited for trials that evaluate aggressive local therapy (eg, escalation of radiation dose). The decision to use aggressive supratotal resection may be justified for prolonged PFS. Alternatively, if simple gross total resection leads to long PFS, physicians may prefer to take the standard surgical approach and avoid the possible risks of supratotal resection if the patient values the quality of life over PFS or OS. Fourth, for long PFS and distant recurrence, additional systemic therapies on trial after standard surgery and chemoradiation therapy could possibly be used to try to prevent or delay the distant recurrence. The benefit of supratotal resection may be limited in these patients.

CaPTk software provides researchers and the clinical community with systematic quantification tools, from image preprocessing to extraction of a comprehensive set of standard radiomic features, including measures of shape and texture that represent the spatial heterogeneity of the tumor landscape. Furthermore, generating predictive models for risk stratification and prognostication could help extract markers that reveal the underlying pathophysiology, including diffusion and perfusion signatures, and identification and integration of the most discriminative features.

PFS is difficult to define; therefore, to address this problem, we tried to standardize the definition using patients with pathologically proven recurrence who had sufficient follow-up scans after initial resection, so that the date of recurrence could be more reliably determined. Our study population was small secondary to a strict inclusion criteria, and future studies with larger prospective cohorts could help with better generalization of the predictive models. In future studies, radiomic models should be compared or added to the predictive models designed on the basis of clinical and/or genomic information for a comprehensive patient prognosis.

In conclusion, our results imply that CIPh signatures that were developed by using radiomic models in CaPTk could predict PFS and RP in patients diagnosed with GBM. These predictions, based on the images acquired before the initial resection, may aid the multidisciplinary neuro-oncology team in planning a more personalized treatment strategy that would then lead to improved outcomes.
